# Photoinduced Electron Transfer in Inclusion Complexes of Carbon Nanohoops

**DOI:** 10.1021/acs.accounts.3c00488

**Published:** 2023-12-16

**Authors:** Olga A. Stasyuk, Alexander A. Voityuk, Anton J. Stasyuk, Miquel Solà

**Affiliations:** Institute of Computational Chemistry and Catalysis and Department of Chemistry, University of Girona, C/ M. Aurèlia Capmany, 69, 17003 Girona, Catalonia, Spain

## Abstract

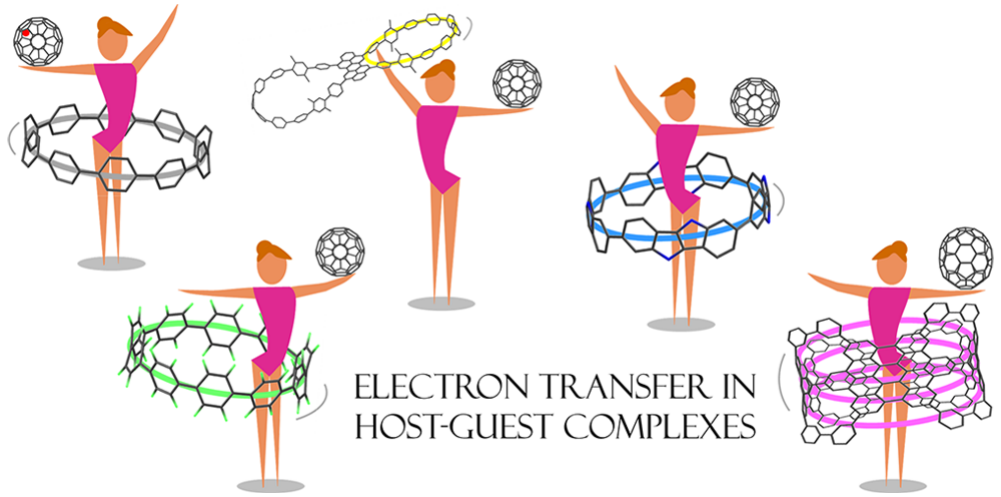

Photoinduced electron transfer (PET) in carbon materials is a process of great importance in light energy conversion. Carbon materials, such as fullerenes, graphene flakes, carbon nanotubes, and cycloparaphenylenes (CPPs), have unusual electronic properties that make them interesting objects for PET research. These materials can be used as electron–hole transport layers, electrode materials, or passivation additives in photovoltaic devices. Moreover, their appropriate combination opens up new possibilities for constructing photoactive supramolecular systems with efficient charge transfer between the donor and acceptor parts. CPPs build a class of molecules consisting of para-linked phenylene rings. CPPs and their numerous derivatives are appealing building blocks in supramolecular chemistry, acting as suitable concave receptors with strong host–guest interactions for the convex surfaces of fullerenes. Efficient PET in donor–acceptor systems can be observed when charge separation occurs faster than charge recombination. This Account focuses on selected inclusion complexes of carbon nanohoops studied by our group. We modeled charge separation and charge recombination in both previously synthesized and computationally designed complexes to identify how various modifications of host and guest molecules affect the PET efficiency in these systems. A consistent computational protocol we used includes a time-dependent density-functional theory (TD-DFT) formalism with the Tamm–Dancoff approximation (TDA) and CAM-B3LYP functional to carry out excited state calculations and the nonadiabatic electron transfer theory to estimate electron-transfer rates. We show how the photophysical properties of carbon nanohoops can be modified by incorporating additional π-conjugated fragments and antiaromatic units, multiple fluorine substitutions, and extending the overall π-electron system. Incorporating π-conjugated groups or linkers is accompanied by the appearance of new charge transfer states. Perfluorination of the nanohoops radically changes their role in charge separation from an electron donor to an electron acceptor. Vacancy defects in π-extended nanohoops are shown to hinder PET between host and guest molecules, while large fully conjugated π-systems improve the electron-donor properties of nanohoops. We also highlight the role of antiaromatic structural units in tuning the electronic properties of nanohoops. Depending on the aromaticity degree of monomeric units in nanohoops, the direction of electron transfer in their complexes with C_60_ fullerene can be altered. Nanohoops with aromatic units usually act as electron donors, while those with antiaromatic monomers serve as electron acceptors. Finally, we discuss why charged fullerenes are better electron acceptors than neutral C_60_ and how the charge location allows for the design of more efficient donor–acceptor systems with an unusual hypsochromic shift of the charge transfer band in polar solvents.

## Key References

- StasyukA. J.; StasyukO. A.; SolàM.; VoityukA. A.Hypsochromic solvent shift of the charge separation band in ionic donor–acceptor Li^+^@C_60_⊂[10]CPP. Chem. Commun.2019, 55, 11195–1119810.1039/c9cc05787k31465052.^1^ In this work, we observed for the first time an unusual effect of destabilization of charge-separated states by polar medium, which leads to a hypsochromic shift of the CT band.
- StasyukA. J.; StasyukO. A.; SolàM.; VoityukA. A.Photoinduced electron transfer in nanotube⊃C_70_ inclusion complexes: phenine vs nanographene nanotubes. Chem. Commun.2020, 56, 12624–12627.10.1039/d0cc04261g32959809(2) This work revealed that the number of vacancy defects strongly affects the rates of charge separation and recombination processes between the host and guest units.
- StasyukO. A.; StasyukA. J.; SolàM.; VoityukA. A.Photoinduced electron transfer in host–guest complexes of double nanohoops. J. Nanostruct. Chem.2022, 10.1007/s40097-022-00518-w.(3) In this work, we described the important role of the linker between two nanohoops for efficient electron transfer in the host–guest complexes of double nanohoops and C_60_ fullerene.
- GeorgeG.; StasyukO. A.; VoityukA. A.; StasyukA. J.; SolàM.Aromaticity controls the excited-state properties of host–guest complexes of nanohoops. Nanoscale2023, 15, 1221–1229.36537223 10.1039/d2nr04037a(4) Studying excited state properties of nanohoops with aromatic and antiaromatic fragments, we found that π-electron delocalization in monomeric units is crucial for directing the electron transfer (from or to nanoring).

## 1 Introduction

**Figure 1:**
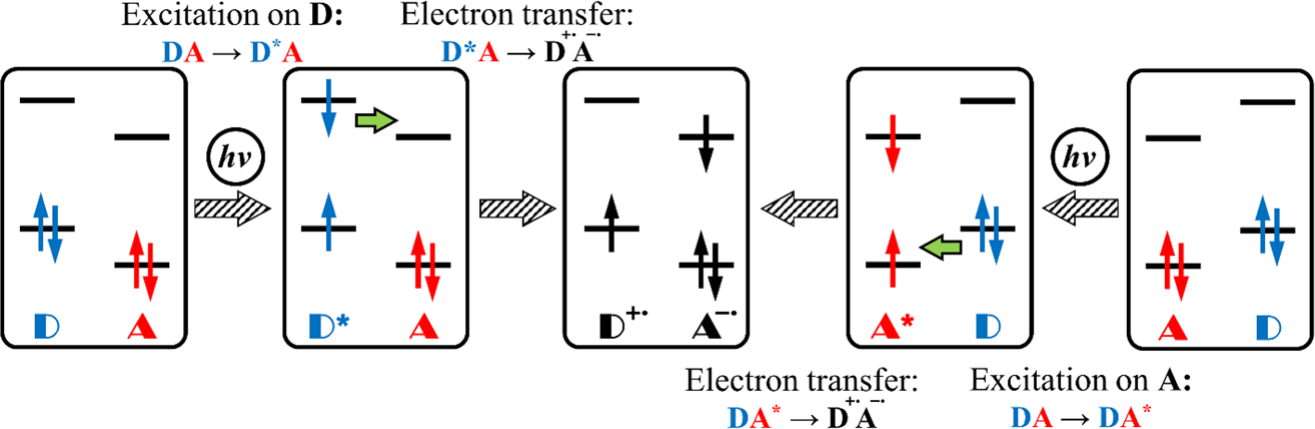
Schematic diagram of PET between the electron donor and electron acceptor.

Photoinduced electron transfer (PET) in complexes is an excited state process that proceeds from the electron donor (**D**) to the electron acceptor (**A**) to generate a charge-transfer (CT) state. This process plays a key role in the solar energy conversion to electricity in photovoltaic cells. The energetics and dynamics of PET are determined by the structures of **D** and **A**, their mutual positions, and the nature of the environment. In PET, a photoexcited state (DA* or D*A) transforms to the ground state (GS) through an intermediate CT state (**D**^**+·**^**A**^**–·**^), and thus it can be viewed as a quenching of the excited state. This process has two possibilities, depending on which fragment (**D** or **A**) undergoes photoexcitation (Figure 1). For PET to occur, **D** and **A** must be close together to allow electron exchange, which requires sufficiently overlapping **D** and **A** wave functions. Thus, to ensure efficient electron transfer, the careful selection of suitable **D** and **A** fragments to establish an effective electronic communication between them is required.

An ideal **DA** system should have a long-lived CT state that forms with a high quantum yield. A molecular spacer between **D** and **A** largely determines the PET dynamics.^5,6^ For this reason, supramolecular systems without spacers, which are assembled through noncovalent interactions, are of particular interest for photovoltaic applications. However, such systems may have poor stability in polar solvents. From this point of view, the concave–convex complementarity turns out to be an excellent strategy to improve their stability. Various macrocyclic host molecules, such as γ-cyclodextrin, butylcalix[8]arene, and cycloparaphenylenes, have been developed for efficient binding of fullerenes. In addition, inclusion complexes can suppress self-aggregation of fullerenes, resulting in improved light conversion efficiency.^7−9^

Cycloparaphenylenes (CPPs) are built from phenylene units linked in *para* positions to form radially π-conjugated molecular loops.^10,11^ They can be considered as the simplest structural unit of armchair carbon nanotubes. Significant developments in organic synthesis allowed the successful isolation and characterization of CPPs containing from 5 to 16 and 18 phenylene units. The CPPs have intriguing size-dependent properties that distinguish them from linear oligoparaphenylenes. In particular, their light absorption remains nearly constant regardless of the nanohoop size, while emission shows a significant red shift as the size decreases. In addition, unlike their linear analogues, the HOMO–LUMO energy gap of CPPs increases with the number of phenyl rings.^12^

Nanohoops are perfect hosts for fullerenes, forming stable host–guest complexes. The first such complex was reported by Yamago and co-workers in 2011.^13^ The diameter of the CPP receptor with 10 phenylene units ([10]CPP) (1.38 nm) is best suited to accommodate C_60_ (0.71 nm), resulting in a stable **[10]CPP⊃C**_**60**_ complex with the binding constant *K*_*a*_ of (2.79 ± 0.03) × 10^6^ L/mol in toluene. Numerous CPP-based inclusion complexes were reported over the past decade, demonstrating the versatility of these systems. The incorporation of π-extended units into nanohoops has notably increased their stability and expanded their potential applications.^14−17^

Given that fullerenes serve as acceptors in organic photovoltaic devices and that CPPs can work as suitable electron donors, their complexes are interesting in terms of charge transfer processes upon light absorption. Calculations indicate that the energetically low-lying transitions in the **[10]CPP⊃C**_**60**_ complex involve intrafullerene charge rearrangements rather than charge transfer between donor and acceptor units.^1,18,19^ As a result, the generation of CT states within this complex is characterized by a positive Gibbs energy. However, notable improvements in the thermodynamics of this process have been observed through specific structural modifications of either CPPs or fullerenes. One of the appealing directions for modulating the electronic properties of CPPs is the inclusion of bridges between phenylene units.^20^

In the last 5 years, we have been working to uncover the key factors affecting the PET processes in the inclusion complexes of carbon nanostructures. Our goal is to computationally design and characterize novel supramolecular complexes that can ultimately serve as competitive replacements for the existing active layers in photovoltaic devices. In this Account, we consider the effects of different structural and electronic factors on PET efficiency:

1. Effects of substituents and linkers in nanohoops.
2. Effects of π-extension and role of vacancy defects.
3. Effects of aromaticity/antiaromaticity.
4. Effects of charge distribution in fullerene-based complexes.

## 2 Methodology

All results discussed below were obtained by using a consistent computational protocol. Electronic structure calculations and vertical excitation energies were calculated using the Tamm–Dancoff approach (TDA)^21^ with the range-separated CAM-B3LYP functional and the def2-SVP basis set.^22,23^ The suitability of this functional for modeling charge-transfer processes in fullerene-based complexes has been demonstrated previously by our group.^24^ For each system, the 80 lowest singlet states were simulated by taking solvent effects into account.

To describe charge and exciton distribution in the ground and excited states, we carried out a quantitative analysis based on the transition density matrix properties.^25^ The excited states were classified into three groups: (1) locally excited (LE) states with the exciton localized on a single fragment and small contribution of charge separation, CS < 0.1***e***; (2) CT states with CS > 0.8***e*** between fragments, and (3) mixed states, where 0.1***e*** < CS < 0.8***e***.

To estimate the rate of nonadiabatic electron transfer between the host and guest, we used a semiclassical method developed by Ulstrup and Jortner.^26^ Since transitions from the GS to CT states usually have a very weak oscillator strength, the CT states are not directly populated because of the negligibly small probability of light absorption. The population of the CT states often occurs as follows: (1) generation of higher LE states due to a stronger oscillator strength of the corresponding transition, (2) rapid dissipation of this state to the lowest LE state through internal conversion, and (3) decay of the lowest LE state into CT states of lower energy by charge separation (electron transfer between the donor and acceptor sites). In our study we primarily focused on step (3), i.e., electron transition between LE and CT states.

Three types of CT can be distinguished in the studied host–guest complexes: (1) from the nanohoop to guest (CT1), (2) from a linker/substituent to guest (CT2), and (3) from guest to nanohoop (CT3). We also accounted for charge recombination, which competes with the charge separation process and recovers the GS of the complex.

## 3 Effects of Substituent/Linker

In 2018, Xu et al. reported a conjugate, in which **[10]CPP** is covalently linked to a zinc porphyrin (ZnP).^27^ The **ZnP-[10]CPP** heterojunction forms an inclusion complex with **C**_**60**_, where the electron donor and acceptor units are well separated. **[10]CPP⊃C**_**60**_ and **ZnP-[10]CPP⊃C**_**60**_ have similar binding constants in toluene, namely, (2.79 ± 0.03) × 10^6^ and (1.6 ± 0.1) × 10^6^ L/mol.^27,28^ Thus, the covalent functionalization of **[10]CPP** by ZnP does not affect the stability of the complex. We then compared the electronic properties of **[10]CPP⊃C**_**60**_ and **ZnP-[10]CPP⊃C**_**60**_.^29^ In both cases, LUMO is localized on fullerene, while HOMO is distributed over **[10]CPP** in **[10]CPP⊃C**_**60**_ and over ZnP in **ZnP-[10]CPP⊃C**_**60**_. Small changes in orbital energies by the formation of the complexes suggest only small charge transfer between the fullerene cage and host unit in the ground state, as was confirmed by the Mulliken population analysis.

**Figure 2:**
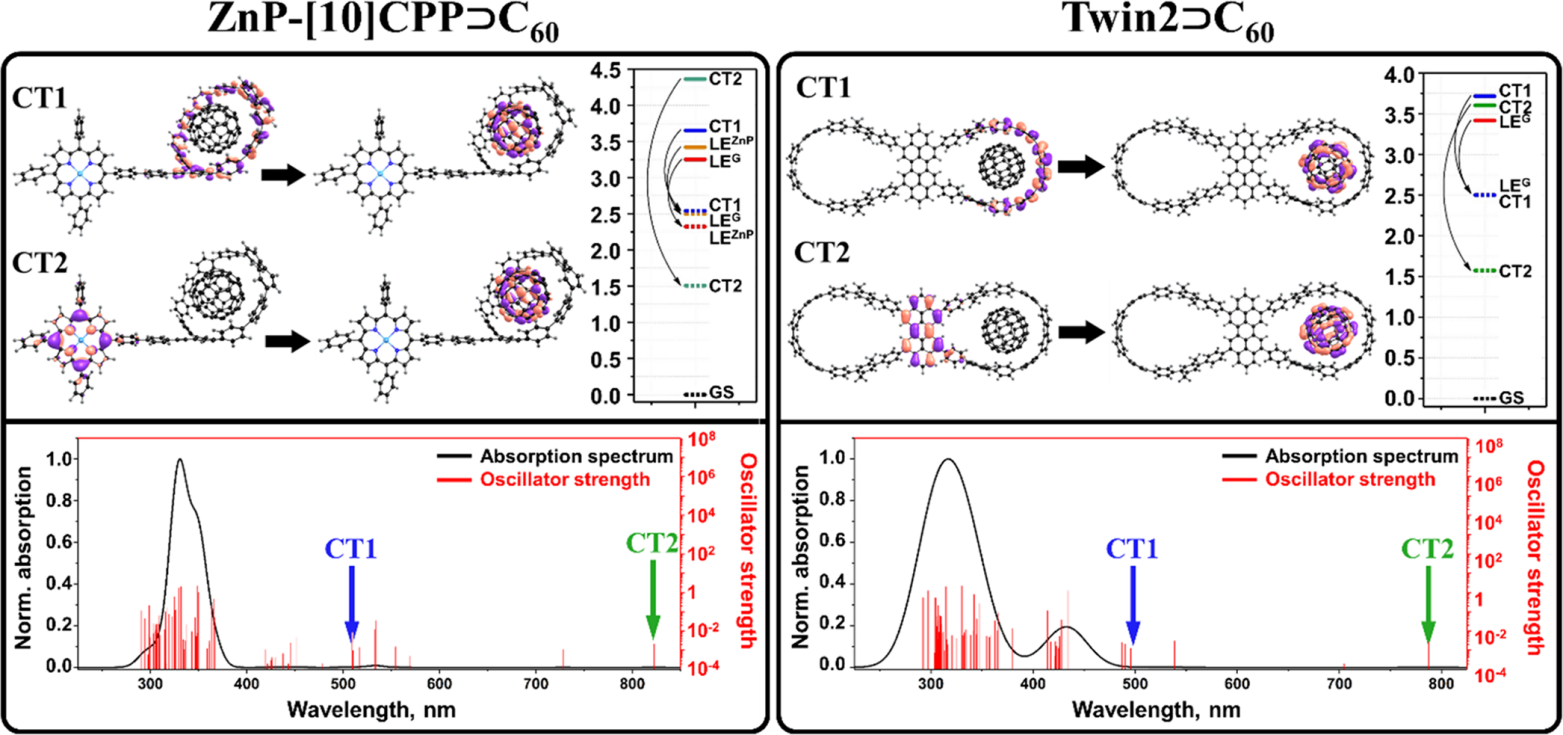
Molecular orbitals representing CT1 and CT2 states, energies of LE and CT states (in eV) in a vacuum (VAC, solid line) and dichloromethane (DCM, dashed line), and UV–vis spectra simulated in DCM for **ZnP-[10]CPP⊃C**_**60**_ and **Twin2⊃C**_**60**_. Adapted with permission from ref (3). Copyright 2022 Springer and from ref (29). Copyright 2020 American Chemical Society.

The lowest LE state in **[10]CPP⊃C**_**60**_ is localized on **C**_**60**_ fullerene (LE^Guest^), while in **ZnP-[10]CPP⊃C**_**60**_, it is localized on the ZnP fragment (LE^ZnP^). The lowest CT states in both complexes correspond to electron transfer (ET) from **[10]CPP** to **C**_**60**_ (CT1). In general, in both complexes, the states of the same nature are characterized by approximately the same energy. This means that a spatially distant ZnP fragment weakly affects the electronic properties of **[10]CPP**. However, for **ZnP-[10]CPP⊃C**_**60**_, an additional type of CT state with ET from ZnP to **C**_**60**_ was found (CT2). The CT2 state is about 0.7 eV higher in energy than CT1. Both CT states are characterized by complete electron transfer (>0.98***e***). Molecular orbitals participating in the CT1 and CT2 states are shown in Figure 2.

Typically, CT states have a large dipole moment, and their energy is more sensitive to a polar medium than the energy of LE or GS states. However, similar dipole moments and weak solvent stabilization were found for the GS and CT1 states. This can be explained by a high symmetry of **[10]CPP** and **C**_**60**_ and their ability to efficiently delocalize the charge. On the contrary, the difference in the dipole moment of the GS and CT2 state in **ZnP-[10]CPP⊃C**_**60**_ is greater than 100 D due to a large distance (∼21 Å) between ZnP^•+^ and **C**_**60**_^•–^, and a strong stabilization of the CT2 state occurs in polar solvents (for example, by 2.2 eV in benzonitrile). Since CT2 is the lowest excited state even in toluene, it is expected to be populated by the decay of LE states and detected in experiment.

In 2021, Du and co-workers reported a double-nanohoop molecule—a highly strained all-phenylene bismacrocycle named conjoined (1,4)[10]cycloparaphenylenophane (**Twin1**).^30^ In addition, π-conjugated frameworks consisting of two CPP units linked by a peropyrene moiety (**Twin2**, Figure 2)^31,32^ and a flexible cyclooctatetrathiophene core (**Twin3**)^33^ were reported. The formation of their inclusion complexes with **C**_**60**_ were confirmed experimentally. In the complexes, HOMO is localized on the nanohoop, while LUMO is localized on the fullerene. The lowest excited states are localized on the fullerene unit (LE^Guest^). The PET properties of the complexes depend on the ring size and linker type of the nanohoop. Two types of CT states were found. Both types are generated by electron transfer from the nanohoop to **C**_**60**_ and can be denoted as **TwinX**^**+•**^**⊃C**_**60**_^–•^.^3^ The CT1 corresponds to an electron transition from the rings of the nanohoop to **C**_**60**_, whereas in the CT2 state an electron is transferred from the linker to **C**_**60**_ (Figure 2). The CT2 state was not found in **Twin1⊃C**_**60**_ within the simulated excited states since the HOMO energy of the benzene linker is significantly lower compared to the HOMOs of peropyrene and cyclooctatetrathiophene.

Interestingly, the CT1 and CT2 states demonstrate different responses to solvation. It can be explained by different changes in the dipole moments during GS → CT1 and GS → CT2 transitions. For CT1 in **Twin2⊃C**_**60**_ and **Twin3⊃C**_**60**_, these differences are 13 and 12 D, respectively. Accordingly, the difference in solvation energies of the CT1 states and GS is small (0.30 to 0.28 eV). In contrast, CT2 is characterized by significantly larger changes in the dipole moment (56.7 and 39.9 D) and by strong solvation energies, namely, −2.03 and −1.54 eV, respectively. Figure 2 shows the energies of the GS, LE^Guest^, and CT states in the gas phase and in dichloromethane (DCM) for **Twin2⊃C**_**60**_. Stabilization of the CT1 state by DCM is sufficient to balance the energies of the LE^Guest^ and CT1 states. In turn, the CT2 state becomes the lowest excited state, lying almost 1 eV lower than LE^Guest^.

The LE^Guest^ → CT1 charge separation process proceeds in the normal Marcus regime (|Δ*G*^0^| < λ) on the subnanosecond time scale (Table 1). In **Twin2⊃C**_**60**_, the LE^Guest^ → CT2 reaction is almost barrierless and also occurs on the subnanosecond time scale. The generation of CT2 in **Twin3⊃C**_**60**_ is endothermic and can hardly be observed.

**Table 1:** Gibbs Energy Difference (Δ*G*^0^) for Denoted Transitions and Charge Separation (*k*_CS_) and Charge Recombination (*k*_CR_) Rates for Selected Inclusion Complexes Calculated in DCM

	Charge separation			Charge recombination		
DA system	Transition	Δ*G*^0^, eV	*k*_CS_, s^–1^	Transition	Δ*G*^0^, eV	*k*_CR_,^a^ s^–1^
**[10]CPP⊃C**_**60**_**-MP**	LE^Guest^ → CT1	0.250	9.17 × 10^5^	CT1 → GS	–2.613	5.16 × 10^0^
**[10]CPP⊃Li**^**+**^**@C**_**60**_**-MP**	LE^Guest^ → CT1	–0.381	1.11 × 10^9^	CT1 → GS	–2.091	4.91 × 10^1^
**PF[10]CPP⊃C**_**60**_	LE^Host^ → CT1	–0.177	1.13 × 10^8^	CT1 → GS	–3.880	2.85 × 10^–13^
	LE^Host^ → CT3	–0.276	1.67 × 10^10^	CT3 → GS	–3.781	1.18 × 10^–9^
**Twin2⊃C**_**60**_	LE^Guest^ → CT1	–0.001	6.21 × 10^10^	CT1 → GS	–2.501	2.10 × 10^4^
	LE^Guest^ → CT2	–0.927	2.06 × 10^10^	CT2 → GS	–1.575	1.80 × 10^11^
**Twin3⊃C**_**60**_	LE^Guest^ → CT1	–0.017	6.46 × 10^10^	CT1 → GS	–2.478	2.25 × 10^4^
	LE^Guest^ → CT2	0.492	2.06 × 10^3^	CT2 → GS	n/a	n/a
**[4]CHBC⊃C**_**70**_	LE^Guest^ → CT1	–0.107	1.29 × 10^11^	CT1 → GS	–2.182	4.91 × 10^2^
**pNT⊃C**_**70**_	LE^Guest^ → CT1	0.798	2.34 × 10^–5^	CT1 → GS	n/a	n/a
**[4]DHPP⊃C**_**60**_	LE^Guest^ → CT1	–0.573	3.60 × 10^10^	CT1 → GS	–1.824	2.35 × 10^7^
	LE^Host^ → CT1	–1.000	3.05 × 10^10^			
**[4]PP⊃C**_**60**_	LE^Guest^ → CT3	–0.615	9.53 × 10^11^	CT3 → GS	–1.790	9.75 × 10^6^
	LE^Host^ → CT3	–0.243	9.58 × 10^12^			

a Reaction takes place in deep inverted Marcus region.

Comparing the results, we draw the following conclusions: (1) the substituent/linker (ZnP, peropyrene, or cyclooctatetrathiophene) does not significantly affect the CT1 state formed after electron transfer from CPP to the fullerene; (2) incorporating these fragments into CPP allows for generation of new CT states, where the fragment acts as an electron donor.

Another way to modify the electronic properties of nanohoops is to add electron-donating or electron-withdrawing substituents without a conjugated π-electron system. In 2022, Shudo et al. succeeded in synthesizing and isolating several perfluorocycloparaphenylenes PF[*n*]CPPs (*n* = 10, 12, 14, 16).^34^

**Figure 3:**
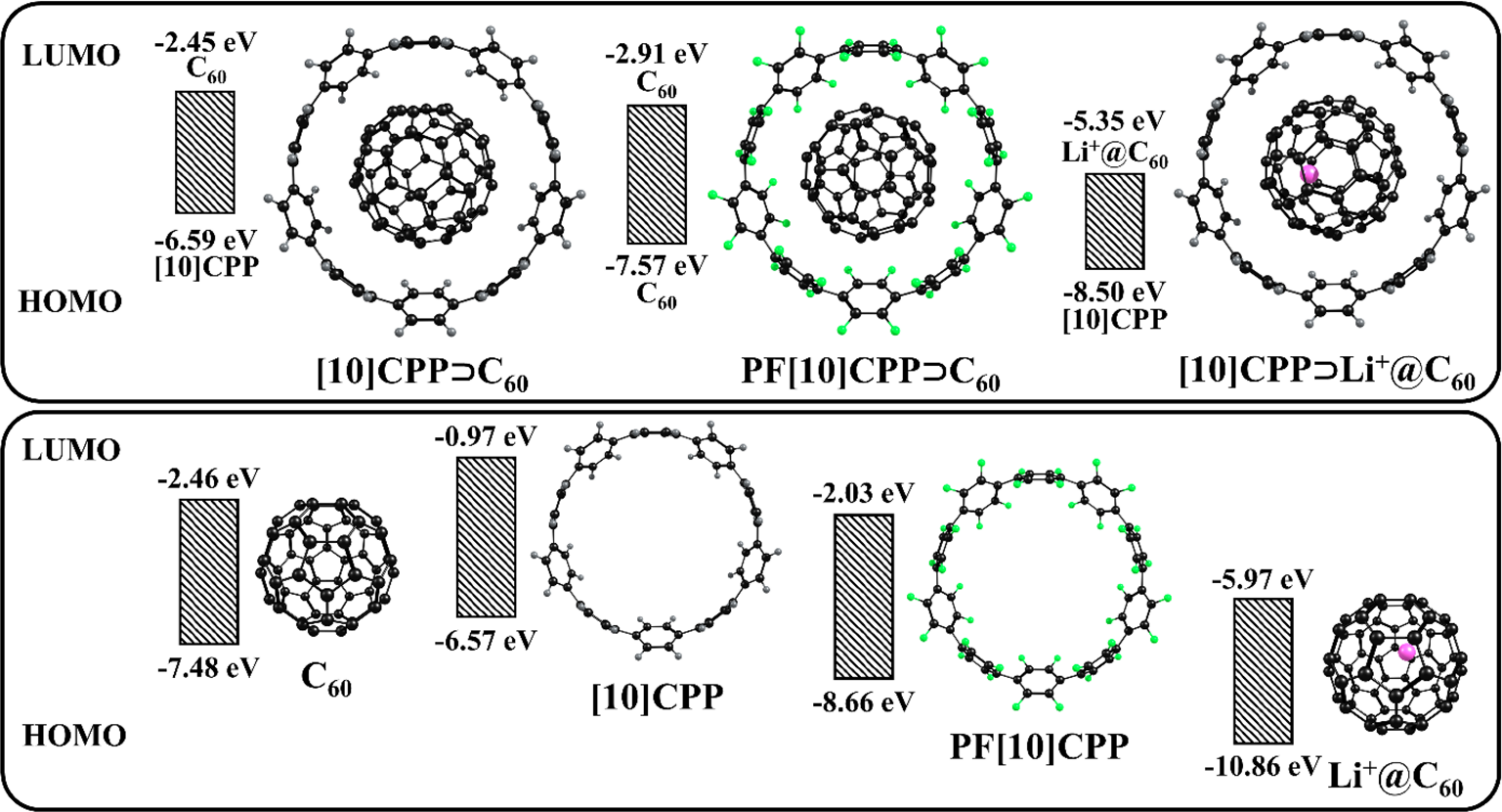
HOMO/LUMO energies of the **[10]CPP⊃C**_**60**_, **PF[10]CPP⊃C**_**60**_, and **[10]CPP⊃Li**^**+**^**@C**_**60**_ complexes and their subunits.

We compared **PF[10]CPP⊃C**_**60**_ with the original **[10]CPP⊃C**_**60**_ to estimate the effect of halogen substituents on the ground and excited state properties of **[10]CPP**.^35^ The noncovalent interactions between the host and guest units in **PF[10]CPP⊃C**_**60**_ was found to be weaker than those in **[10]CPP⊃C**_**60**_ due to reduced π–π interactions resulting from an increased dihedral angle between phenyl rings, a consequence of replacing hydrogen atoms with fluorine atoms. The electronic properties of **[10]CPP** are significantly altered by the fluorine substituents, lowering its HOMO by about 2 eV and LUMO by about 1 eV in **PF[10]CPP** (Figure 3).

Although **PF[10]CPP⊃C**_**60**_ and **[10]CPP⊃C**_**60**_ have similar structures, their electronic properties differ significantly. The HOMO of **[10]CPP⊃C**_**60**_ is localized on the host unit, while the HOMO of **PF[10]CPP⊃C**_**60**_ is localized on guest **C**_**60**_. Due to its low-lying HOMO, **PF[10]CPP** cannot act as an electron donor like **[10]CPP** in complexes with **C**_**60**_. However, it can work as an electron acceptor due to its low-lying LUMO.

**[10]CPP⊃C**_**60**_ has only one type of CT among the 80 lowest singlet excited states: **[10]CPP**^**+•**^**⊃C**_**60**_^**–•**^, whereas **PF[10]CPP⊃C**_**60**_ has two types of CT states. The CT1 state, **PF[10]CPP**^**+•**^**⊃C**_**60**_^**–•**^, is similar to the CT state in **[10]CPP⊃C**_**60**_, while the CT3 state, **PF[10]CPP**^**–•**^**⊃C**_**60**_^**+•**^, is generated by electron transfer from **C**_**60**_ to **PF[10]CPP**. Importantly, the energy of CT3 is 0.3 eV lower than that of CT1. The CT1 state is characterized by almost complete charge transfer (CT = 0.95***e***), while in the CT3 state only 0.80***e*** is transferred. This, in turn, leads to a stronger stabilization of the CT1 state by polar media compared to CT3.

In **PF[10]CPP⊃C**_**60**_, the strongly absorbing transitions mostly occur on the host unit. Therefore, the main pathway for generating CT states is the decay of the LE^Host^ state. The generation of CT3, when **C**_**60**_ is an electron donor, is about 2 orders of magnitude faster than the generation of CT1, where **C**_**60**_ is an electron acceptor. In turn, charge recombination takes place in the inverted Marcus region, and it is much slower than charge separation. Moreover, the exciton transfer rate between the LE^Host^ and LE^Guest^ states is 1.0 × 10^5^ s^–1^. Thus, the processes of recombination and exciton transfer will not compete with PET.

In summary, multiple substitutions with electron-withdrawing substituents are highly promising. The replacement of hydrogen with fluorine atoms converts electron donor **[10]CPP** into an electron acceptor. The inclusion complex of **PF[10]CPP** exhibits a unique feature, electron transfer from **C**_**60**_ to the host molecule.

## 4 Effects of π-Extension

As mentioned above, decorating nanohoops with π-extended fragments is an efficient way to generate new types of CT states. In turn, an extension of the π-electron systems of nanohoops can strongly affect their properties, in particular, increase their electron-donor characteristics. An example is the oligomer **([10]CPP_Fused)**_**n**_—an appealing host unit with extended π-conjugation.^36^ It forms stable complexes with fullerene that exhibit efficient electron transfer from the host to guest molecules both within the same monomer unit and between different units.

**Figure 4:**
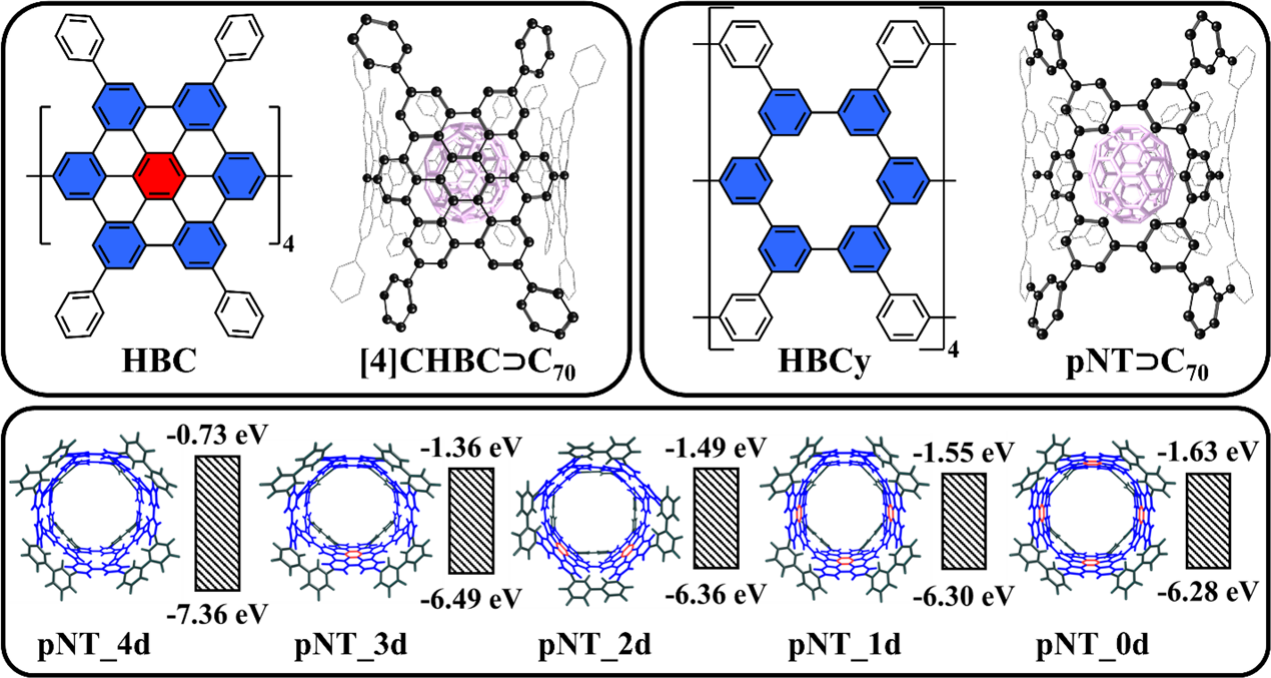
Graphical representation of **[4]CHBC⊃C**_**70**_ and **pNT⊃C**_**70**_, and HOMO/LUMO energies of phenine nanotubes with defects. Adapted with permission from ref (38). Copyright 2021 Wiley VCH.

In 2017, Du and co-workers synthesized a π-extended carbon nanohoop based on hexa-peri-hexabenzocoronene (HBC). The cyclic tetramer (**[4]CHBC**) has a diameter similar to **[12]CPP** and can form a stable 1:1 inclusion complex with **C**_**70**_ fullerene, with a *K*_*a*_ of 1.07 × 10^6^ L/mol in toluene.^17^ Besides, there are structural analogues of **[4]CHBC** known as phenine nanotubes (**pNTs**).^37^ They consist of four hexabenzenacyclohexaphane units, which are HBC fragments with six carbon-atom vacancy defects in the center (Figure 4).

The electronic structure of **pNT** differs significantly from **[4]CHBC**. Compared to **[4]CHBC**, its HOMO is about 1 eV lower but its LUMO is 0.9 eV higher.^2^ We studied a series of phenine nanotubes with different numbers of vacancy defects, **pNT_*x*d** (*x* = 0, 1, 2, 3, 4).^38^**pNT_4d** has four defects, **pNT_3d** has one less defect, etc. The number of defects decreases by one in each subsequent model until **pNT_0d**, which comprises four HBC units and is defect-free. As the HOMO energy decreases with the number of defects but the LUMO energy increases, the HOMO–LUMO gap changes from 4.61 eV in **pNT_0d** to 6.63 eV in **pNT_4d**. The lower HOMO energy of **pNT_4d** indicates significantly poorer electron-donor properties compared to those of **[10]CPP** and **[4]CHBC**. Interestingly, the **pNT** length has a minor effect on the electron-donating properties. In particular, the HOMO energy changes only within 0.1 eV by lengthening the **pNT_0d** model from 264 to 504 carbon atoms.

In all host–guest complexes, the LUMO is localized on **C**_**70**_, while the HOMO is localized on the host unit. The lowest excited state is LE^Guest^, with an energy ranging from 2.20 to 2.30 eV. The CT1 state has a higher energy, which strongly depends on the number of vacancy defects in **pNT** and ranges from 3.49 eV for **pNT_4d⊃C**_**70**_ to 2.44 eV for **pNT_0d⊃C**_**70**_. Thus, the electron-donor ability of the host unit increases with a decrease in the number of defects.

The stabilization of the CT1 state by DCM is not sufficient to reorder the CT1 and LE^Guest^ states in **pNT_4d⊃C**_**70**_. However, DCM solvation plays an important role in complexes containing at least one HBC unit (without vacancy defects) in **pNT** and in **[4]CHBC⊃C**_**70**_. The gap between the CT1 and LE^Guest^ states varies from 0.06 eV in **pNT_3d⊃C**_**70**_ to −0.11 eV in **[4]CHBC⊃C**_**70**_, allowing for the efficient population of the CT1 state through the decay of the LE^Guest^ states. In **[4]CHBC⊃C**_**70**_, this process occurs on the picosecond time scale with *k*_CS_ of 1.29 × 10^11^ s^–1^. The high positive Gibbs energy found for charge separation in **pNT_4d⊃C**_**70**_ makes PET unlikely to occur in this complex. For the other complexes of **pNT**, charge separation occurs in the normal Marcus region on the subnanosecond time scale, while charge recombination takes place in the deeply inverted Marcus region and is much slower. The rates of both CS and CR depend on the number of vacancy defects in **pNTs**. The CS rate remains in a narrow range and varies from 8.6 × 10^9^ to 8.5 × 10^10^ s^–1^, but the CR rate sharply decreases as the defects disappear. For instance, in **pNT_3d⊃C**_**70**_, with a single HBC unit, the CS rate is 8.5 × 10^9^ s^–1^ and the CR rate is 1.8 × 10^7^ s^–1^. In this complex, charge recombination can be an effective channel to deactivate the CT state and hinder the separation of ion pairs over long distances. In contrast, the CS process in **pNT_0d⊃C**_**70**_ is fast (8.5 × 10^10^ s^–1^) but the CR reaction is very slow (3.1 × 10^2^ s^–1^). Thus, controlling the number and position of these defects in π-extended nanohoops can be used to fine-tune their photophysical properties.

## 5 Effects of Aromaticity/Antiaromaticity

Until recently, only nanohoops with aromatic subunits were known. However, in 2020, Esser and co-workers succeeded in incorporating two antiaromatic dibenzo[*a,e*]pentalene (DBP) units into **[12]CPP**.^39^ Later, they reported nanohoops made solely from DBP units—[*n*]DBP, where *n* = 4, 5.^40^ The size and round shape of [4]DBP allow for efficient accommodation of **C**_**60**_ with a binding constant of (1.35 ± 0.03) × 10^5^ L/mol in toluene.

**Figure 5:**
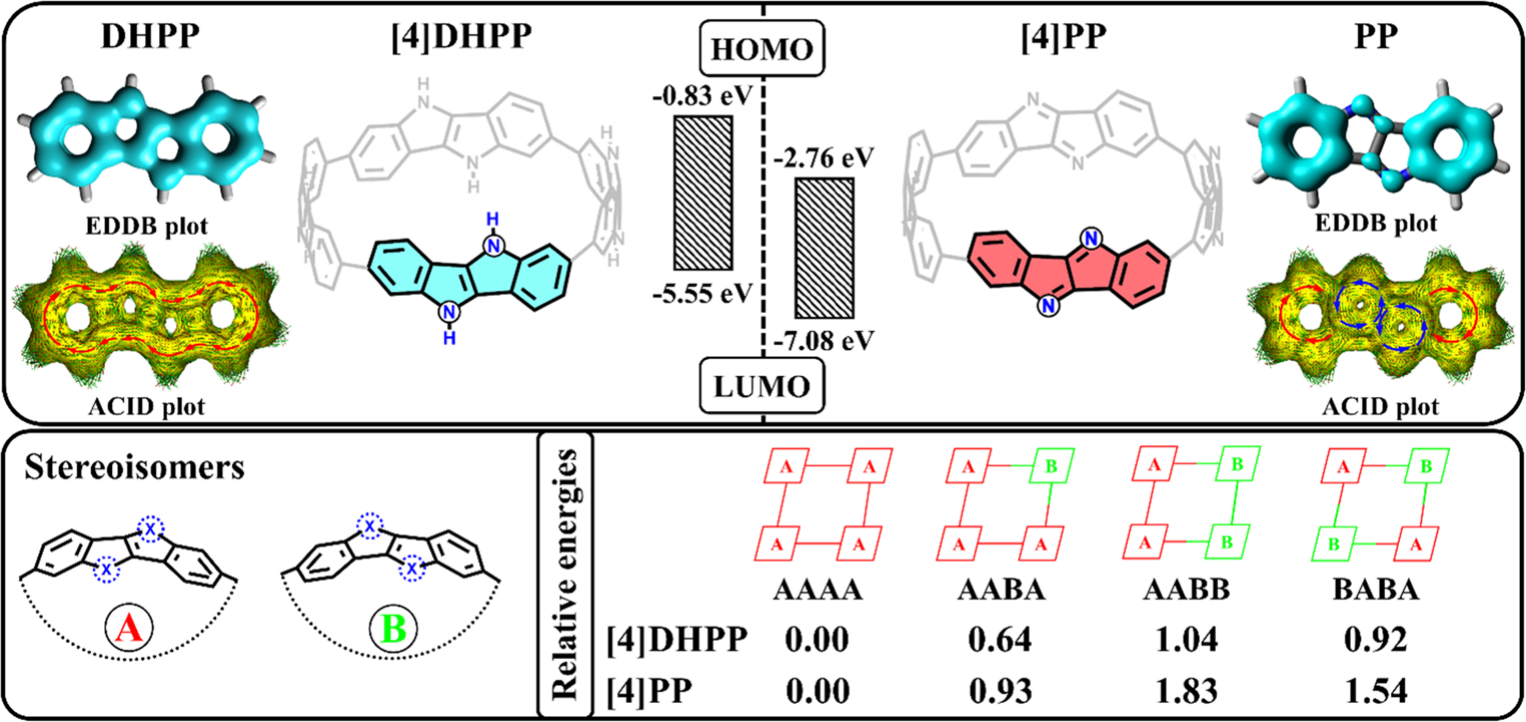
π-EDDB and ACID plots for **DHPP** and **PP** monomers, and structure and relative energy (in kcal/mol) of **[4]DHPP** and **[4]PP** stereoisomers. Adapted with permission from ref (4). Copyright 2023 Royal Society of Chemistry and from ref (41). Copyright 2023 Wiley VCH.

We considered two nanohoops built from benzene-fused antiaromatic pyrrolo[3,2-*b*]pyrrole (**PP**) and its aromatic analogue 1,4-dihydropyrrolo[3,2-*b*]pyrrole (**DHPP**) as host molecules (Figure 5).^4^ The antiaromatic nanohoop has a HOMO–LUMO gap that is 0.4 eV smaller than its aromatic analogue. This difference in orbital energies results in a distinct electronic nature of their complexes with fullerene. In particular, in **[4]DHPP⊃C**_**60**_, LUMO is located on **C**_**60**_, while HOMO is distributed over **[4]DHPP**. Conversely, in **[4]PP⊃C**_**60**_, LUMO is located on the nanohoop, and HOMO is on **C**_**60**_.

In the gas phase, the lowest excited state of **[4]DHPP⊃C**_**60**_ is the CT1 state (at 1.85 eV) with the expected electron transfer from **[4]DHPP** to **C**_**60**_. The LE^Guest^ and LE^Host^ states have energies higher than CT1. In turn, the lowest excited state of **[4]PP⊃C**_**60**_ is the LE^Host^ state. As expected, the LE^Guest^ energy is almost the same in both systems. A key finding in **[4]PP⊃C**_**60**_ is the existence of an unusual electron transfer from the fullerene to the nanohoop. The generated CT3 state has a much lower energy than the CT1 state with electron transfer from **[4]PP** to **C**_**60**_. A comparison of aromaticity descriptors (EDDB and HOMA) for **[4]PP** in neutral, cationic, and anionic forms revealed that the removal of an electron and the generation of **[4]PP**^**+•**^ reduces the aromaticity of nanohoops, while the formation of **[4]PP**^**–•**^ enhances the delocalization of π-electrons in the tetramer. Thus, the higher stability of the CT3 state is caused by an increase in the aromaticity of **[4]PP**. The COSMO solvation model shows a slight stabilization of the CT states in DCM due to the π-extended character and high symmetry of the systems. In **[4]DHPP⊃C**_**60**_, the CT1 state remains the lowest excited state. In **[4]PP⊃C**_**60**_, the CT1 energy is too high and cannot be sufficiently stabilized by DCM. As a result, the CT3 state becomes the lowest excited state in DCM.

The modeled **[4]DHPP** and **[4]PP** nanohoops can exist in several diastereomeric forms, which are achieved by the rotation of one or more monomer units around single C–C bonds. We considered four stereoisomers of each nanohoop and found that the most symmetric isomer (AAAA in Figure 5) is the most stable.^41^ However, all isomers are found within a narrow energy range (less than 2 kcal/mol), suggesting that all of them could be present in the reaction mixture. The calculated energy barriers for the rotation of a monomer unit around the C–C bond are relatively high, 26.3 and 28.5 kcal/mol at the CAM-B3LYP-D3(BJ)/def2-TZVP level for **[4]DHPP** and **[4]PP**, respectively. Therefore, the formation of specific isomers is mainly determined by the synthetic path. The HOMO and LUMO energies of **[4]DHPP** vary noticeably among stereoisomers. As a consequence, the energy of its CT state varies with that of the isomer. The most symmetric isomer has the smallest HOMO–LUMO gap, implying slightly better electron-donor properties. In contrast, **[4]PP** isomers do not show significant changes in orbital energies. Thus, the excited state energy characteristics for all of the studied **[4]PP⊃C**_**60**_ stereoisomers are very similar.

Charge separation in **[4]DHPP⊃C**_**60**_ has a negative Gibbs energy (Table 1). The rates of the CT1 state generation from LE^Guest^ and LE^Host^ are 3.60 × 10^10^ and 3.05 × 10^10^ s^–1^, respectively. In turn, the CR reaction (CT1 → GS) is 3 orders of magnitude slower than CS. The calculated rates depend weakly on the specific **[4]DHPP** stereoisomer. For **[4]PP⊃C**_**60**_, the generation of the CT1 state from LE^Guest^ and LE^Host^ is unlikely due to its positive Gibbs energy and high activation energy. However, the formation of CT3 from the LE states has low activation energies and proceeds on the picosecond time scale. The relatively slow charge recombination CT3 → GS reaction suggests a long lifetime for the CT3 state.

Thus, π-electron delocalization in the monomers controls the electron transfer (from or to nanohoop). The antiaromatic **[4]PP** has significantly lower HOMO and LUMO energies compared to **[4]DHPP**, which improves their electron-acceptor but degrades their electron-donor properties.

## 6 Effects of Guest Charge Distribution

Electron transfer in complexes of nanohoops can also be facilitated by modifying the fullerene cage. One of the most effective approaches to improve the electron-acceptor properties of fullerene is its doping with a Li^+^ ion.

Following the syntheses of **[10]CPP⊃Li**^**+**^**@C**_**60**_(28) and **ZnP-[10]CPP⊃Li**^**+**^**@C**_**60**_,^27^ we studied their excited state properties and compared them with the properties of **[10]CPP⊃C**_**60**_ to reveal the influence of Li^+^ on PET.^1,29^

The insertion of Li^+^ does not change the HOMO and LUMO locations but affects the HOMO–LUMO gap (Figure 3), reducing it from 4.15 eV in **[10]CPP⊃C**_**60**_ to 3.15 eV in **[10]CPP⊃Li**^**+**^**@C**_**60**_. The HOMO and LUMO energies of the Li^+^-doped **C**_**60**_ are lower by about 3 eV than those of the empty fullerene due to the electrostatic potential of the Li^+^ cation. The low-lying LUMOs make the Li^+^-doped fullerene a better electron acceptor than the empty **C**_**60**_, facilitating electron transfer in its complexes.

The energies of the LE^Host^ and LE^Guest^ states show little sensitivity to the Li^+^ encapsulation. In turn, the CT1 state generated by electron transfer from **[10]CPP** to **Li**^**+**^**@C**_**60**_ is more than 1 eV lower compared with the neutral complex and becomes the lowest excited state. This finding is consistent with spectroscopic measurements that revealed the appearance of a new absorption band around 700 nm for cation-doped complexes.^28^ This new band was observed not only for the Li^+^-doped complex but also for complexes with other alkali metal cations.^18^

**Figure 6:**
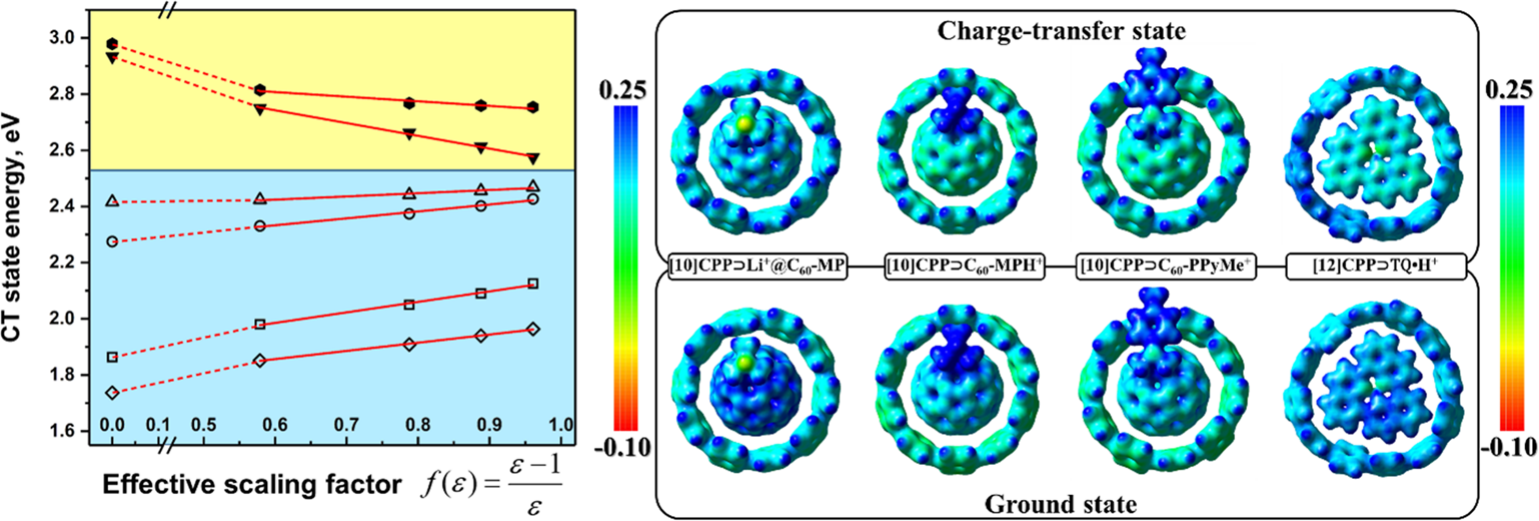
Dependence of CT1 state energy on solvent polarity for **[10]CPP⊃C**_**60**_**-MP** (▼), **[10]CPP⊃Li**^**+**^**@C**_**60**_**-MP** (□), **[10]CPP⊃C**_**60**_**-MPH**^**+**^ (○), **[10]CPP⊃C**_**60**_**-PPyMe**^**+**^ (Δ), **[12]CPP⊃TQ•H**^**+**^ (⬢), and **ZnP-[10]CPP⊃Li**^**+**^**@C**_**60**_ (◇) and selected MEP surfaces in the GS and CT states. Adapted with permission from ref (19). Copyright 2021 Wiley VCH.

The most striking result is the response of the CT states to the solvent polarity. The CT1 state of the neutral **[10]CPP⊃C**_**60**_ complex is more stable in polar solvents, leading to a bathochromic (red) shift of its CT band with increasing solvent polarity. However, the CT1 state of the Li^+^-doped complex is more stable in nonpolar solvents, causing a hypsochromic (blue) shift of its CT band (Figure 6). Furthermore, two CT bands in **ZnP-[10]CPP⊃Li**^**+**^**@C**_**60**_ exhibit the opposite dependence on the solvent polarity. In particular, CT1 shows a rarely observed hypsochromic shift, while CT2 shows a bathochromic shift. Thus, the population of the CT states can be controlled by changing the solvent. In nonpolar media, only CT1 states can be populated by the decay of the LE^Guest^ states. However, in polar media, the probability of CT2 generation increases.

To explore the blue shift of the CT1 band in charged complexes, fulleropyrrolidine derivatives were studied with the positive charge located inside the fullerene cage, near the cage, and shifted away from the cage, namely, **[10]CPP⊃Li**^**+**^**@C**_**60**_**-MP** (MP = *N*-methylfulleropyrrolidine), **[10]CPP⊃C**_**60**_**-MPH**^**+**^, and **[10]CPP⊃C**_**60**_**-PPyMe**^**+**^ (PPyMe = *N*-methylpyridiniumfulleropyrrolidine).^19^ In the complexes, the HOMO is localized on **[10]CPP**, while the LUMO is localized on the fullerene. Going from **[10]CPP⊃C**_**60**_**-MP** to its Li^+^-doped analogue, the HOMO–LUMO gap decreases from 4.21 to 3.31 eV. This change in the energy gap becomes smaller as the positive charge moves away from the center of the complex.^19^ All charged species are significantly better electron acceptors than neutral fullerene. Consequently, the CT1 states formed by electron transfer from **[10]CPP** to the fullerene moiety are the lowest excited states in all cases. The introduction of a positive charge does not affect the energy of the LE states but strongly stabilizes the CT states.

Molecular electrostatic potential (MEP) analysis shows that the hypsochromic shift of the CT band is caused by MEP changes in the guest moiety. The magnitude of this shift is inversely proportional to the distance from the positive charge to the center of the complex. In **[10]CPP⊃Li**^**+**^**@C**_**60**_**-MP**, where the charge is almost at the center of the complex, the hypsochromic shift is maximal (0.23 eV). However, this effect quickly disappears as the charge becomes accessible to the solvent. In **[10]CPP⊃C**_**60**_**-PPyMe**^**+**^, the shift is only 0.04 eV. Surprisingly, we found a red solvatochromic shift for the CT band in **[12]CPP⊃TQ•H**^**+**^ in contrast to the blue shift seen in **[10]CPP⊃Li**^**+**^**@C**_**60**_.^42^ This dissimilarity can be attributed to the increased accessibility of the encapsulated **TQ•H**^**+**^ fragment for solvent molecules and a distortion of **[12]CPP**.

Charge separation in neutral **[10]CPP⊃C**_**60**_**-MP** is characterized by a positive Δ*G*^0^ value, and thus PET is unlikely to be observed in this system (Table 1). In turn, for all charged complexes the PET process is fast, with the characteristic time ranging from nanoseconds (for **[10]CPP⊃Li**^**+**^**@C**_**60**_**-MP**) to picoseconds (for **[10]CPP⊃C**_**60**_**-MPH**^**+**^ and **[10]CPP⊃C**_**60**_**-PPyMe**^**+**^).

These results indicate that introducing a charge on the fullerene significantly improves its electron-acceptor properties and facilitates charge separation in the inclusion complexes. Moreover, the CT bands of such complexes demonstrate a hypsochromic shift in polar solvents.

## 7 Conclusion and Outlook

In this Account, we have summarized the effects of structural modifications on the parameters of photoinduced electron transfer in the inclusion complexes of carbon nanohoops, investigated in our group over the last five years.

Carbon nanohoops have attracted attention because of their tunable size and their ability to form host–guest complexes with fullerenes. The diversity of their supramolecular complexes, which feature unique topology and electronic properties, is constantly growing due to recent advances in organic synthesis. Playing with the nanohoop structure, including incorporation of π-conjugated fragments, multiple fluorine substitutions, extension of the shared π-electron system, and introduction of antiaromatic units, allows for modification of their electronic and photophysical properties. For example, π-conjugated substituents or linkers can act as electron donors instead of a nanohoop, transferring electrons from the substituent/linker to the guest unit. Extension of the conjugated structure of the nanohoop improves its electron-donor properties and facilitates PET from nanohoop to guest. On the other hand, perfluorination of the nanohoop and addition of antiaromatic structural units completely alter its electronic properties, converting the nanohoop from an electron donor to an electron acceptor. Another powerful strategy for tuning the photophysical properties of nanohoops is the control of the number and location of vacancy defects within their π-electron system. While fully conjugated structural motifs improve the donating properties of nanohoops, vacancy defects hinder PET between the host and guest molecules. Moreover, modification of the fullerene guest also plays an important role in the excited state processes occurring in host–guest complexes. A positively charged fragment stabilizes the LUMO of fullerene, making the charged fullerenes better electron acceptors compared to the neutral cages and facilitating PET from the nanohoop to fullerene.

It is expected that the results collected in this Account will be of interest to chemists specializing in the development of new, as yet unexplored, carbon-based supramolecular complexes. One of the exciting directions for further research in this area is the use of polymer chains of carbon nanostructures as the host, which act as either electron donor or acceptor, with the guest molecules having opposite electronic properties. Extended π-conjugation within the host unit ensures high carrier mobility through the material, while strong host–guest interactions guarantee the formation of an ordered structure with well-distributed donor and acceptor units for efficient charge separation.
